# “*A frightening experience, especially at our age”*: Examining the neglect and abuse of older persons in HIV prevention and care programs

**DOI:** 10.3389/fpubh.2023.1061339

**Published:** 2023-03-13

**Authors:** Makandwe Nyirenda, Sizakele Sukazi, Cebo Buthelezi, Jill Hanass-Hancock

**Affiliations:** ^1^Burden of Disease Research Unit, South African Medical Research Council, Cape Town, South Africa; ^2^College of Health Sciences, School of Nursing and Public Health, University of KwaZulu-Natal, Durban, South Africa; ^3^HIV Prevention Research Unit, South African Medical Research Council, Durban, South Africa; ^4^Gender and Health Research Unit, South African Medical Research Council, Durban, South Africa; ^5^College of Health Sciences, School of Health Sciences, University of KwaZulu-Natal, Durban, South Africa

**Keywords:** neglect, discrimination, stigma, disrespect, abuse, HIV, older persons, South Africa

## Abstract

**Background:**

As the global population ages and the HIV pandemic matures, a growing number of older persons aged ≥50 years are becoming increasingly vulnerable to acquiring HIV. Unfortunately, older persons are often neglected and left out of sexual health programs and services. This study explored the experiences of older persons living with and without HIV in accessing prevention and care services and how those experiences translate into the neglect and abuse of older persons. The study also explored older people's perspectives on community responses to HIV in older people.

**Methods:**

This qualitative analysis used data from 37 individuals who participated in focus group discussions conducted in 2017/2018 across two communities in Durban, South Africa. Using an interview guide and thematic content analysis, salient themes regarding attitudes to HIV in older persons and factors of access to HIV prevention and cares services for older persons were analyzed.

**Results:**

The study participant's mean age was 59.6 years. Major themes emerging from the data included factors impacting HIV prevention and transmission in older people; community responses to HIV contributing to the abuse of older people, and structural drivers of abuse in older people living with HIV (OPLHIV). Knowledge about HIV and how to protect themselves from HIV was limited among participants. Older persons were frightened to acquire HIV at an older age as they feared judgment and discrimination for getting HIV at that age. OPLHIV reported frequently experiencing community stigma and poor staff attitudes and practices at health facilities, such as a triage health delivery system that fueled community stigma. Participants also experienced neglect, verbal and emotional abuse at healthcare facilities.

**Conclusion:**

Although there were no reports of physical and sexual abuse of older persons in this study, this study shows that despite decades of HIV programs in the country, HIV-related stigma, discrimination, and disrespect of older persons remain pervasive in the community and at health facilities. As an increasing number of people age and live longer with HIV, the neglect and outright abuse of older persons need urgent policy and program interventions.

## Background

As the global population ages rapidly (1), there are millions of older people vulnerable to age-related health problems including HIV (2, 3). The 2022 United Nations Population estimates show that currently, about 10% (771 million persons) of the global population is defined as “aged” (defined as persons aged 65 years or older) (1). The UN report estimates that the “aged” proportion will reach 16% (over 1.5 billion persons) by 2050. Countries in sub-Saharan Africa are expected to contribute more than half of the projected increase in the global population due to the momentum of past high fertility rates and a young age structure (1). At a national level, rapid growth in the “aged” population impacts efforts toward the attainment of Sustainable Development Goals (SDGs), especially those pertaining to health and social wellbeing. At an individual level, a growing number of older persons face health conditions that need to be managed. In the absence of inclusive health systems, they may also face the risk of being neglected or ill-treated at the health facility level (4–6).

Persons as they age expect to be cared for by their children, other relatives, or in care facilities in the absence of familial care when they cannot care for themselves anymore (7). However, this expected care is not always given or may be rendered in such a way that causes distress or harm to the older person (8). In recent times, economic pressures have also increased, particularly in Africa, making it more difficult for families or social systems to provide care, increasing the risk of neglect or abuse in older persons (9–11). The WHO defines any action or lack thereof in any relationship where trust is expected that distresses or causes harm to an older person as elder abuse (4). Elder abuse (EA) can occur in many forms, including physical, sexual, economic, and emotional abuse as well as exploitation (6). Communities and health facilities are some of the places where elder abuse in the form of disrespect and dehumanizing treatment of older persons takes place (12–14). In this context, some healthcare needs are more neglected than others. According to the United Nations, older persons, alongside persons with disabilities and indigenous persons, often face exclusion and marginalization in sexual and reproductive health rights access (15). On the one hand, older persons' sexual and reproductive health needs might be neglected, while on the other hand, those living with HIV may face a lack of access to care and treatment, increasing both groups' vulnerability to poor health outcomes (16). EA itself has health implications and has been found to be associated with a greater risk for multiple morbidities, including mental health and malnutrition (17, 18), pneumonia, chronic musculoskeletal disorders, and stroke (6, 19). EA, therefore, can result in greater need for health services as well as hospital admission and re-admission (20, 21). Neglected and abused older persons are also about twice as likely to die as older persons who are not neglected or abused (22, 23).

Evidence shows that an increasing number of adults aged 50 years and over are living with HIV (24). This increase in older adults living with HIV is a result of increased survival of persons who acquired HIV under 50 years and are on antiretroviral therapy (ART) (25) and those who newly acquire HIV in their 50s. It was estimated that the number of older people aged 50 years and over living with HIV globally would increase from 5.7 million (16%) in 2016 to 7.5 million (21%) in 2020 (26). Eastern and Southern Africa would be the epicenter of the epidemic accounting for nearly two-thirds of the global population aging with HIV. It has been shown in the literature that the experience of HIV in older persons is unlike in younger persons. Older persons are known to find HIV acquisition more distressing and encounter more difficulties with disclosure and accessing HIV care (27–30). The link between HIV and ageist stereotypes has been well established in the literature (31, 32). This is despite the major risk factors for HIV being similar for young and older people. As such, the American Centers for Disease Control and Prevention (CDC) recommends that all persons aged 13–64 years be tested for HIV at least once during routine healthcare (33). However, due to a perceived low HIV risk perception in older people (34), a lack of age-appropriate HIV prevention messages (35), and healthcare worker attitudes, older people seeking HIV services are often neglected and abused (36).

However, data on the neglect and abuse of older persons in accessing HIV prevention, care, and treatment services, especially from sub-Saharan African settings, are scarce. Despite the growing population of older persons in the population globally and locally as well as the increase in the number aging with HIV, there has been limited research and policy prioritization of the neglect and abuse of older people in the healthcare system (15, 37, 38). Some studies, albeit small and localized, suggest elder abuse in South Africa could be as high as 60% in men and women (39). Our aim was, therefore, to explore the experiences of older persons living with and without HIV in accessing HIV prevention and care services and how those experiences may translate into the neglect and abuse of older persons. In addition, this study explored older person's perspectives of community responses to HIV in older persons. The rapid aging of the population and a growing number of persons aging with HIV requires an understanding and appropriate response to the experiences of older persons in general accessing health services as well as the experiences of older persons living with HIV.

## Methods

### Study setting and population

This study was conducted among community-dwelling older adults from Durban KwaZulu-Natal (KZN), South Africa. KZN is the second most populated area in the country with the population distributed across urban, peri-urban, and rural areas in formal and densely populated informal settings (40). KZN as a province has the highest adult HIV rates in the country with an estimated prevalence of 18.1% (41) and an incidence rate of 1.96 per 100 person-years (95% confidence interval (1.62–2.37) (42). Data from a rural South African population estimated HIV prevalence in 2008 at 9.5% (95% confidence interval, 8.4–10.7) (43). While estimates from the United Nations showed that by 2014 over 2 million or 13% of all adults living with HIV in sub-Saharan Africa were aged 50 years and above (44). Increasing life expectancy, successes of the HIV antiretroviral treatment program, and declining HIV incidence in young adults (44–46), in addition to risky sexual behavior among older people and the phenomenon of intergenerational sexual relationships (47–49), are some of the key contributing factors to an increasing number of older adults living with HIV. Thus, KZN as the epicenter of the HIV pandemic and an increasingly aging population was purposively selected as the setting for this study.

In contrast to the demographic definition of the aged population as 65 years and above (1), we adopted the widely used convention in HIV-related research to classify persons aged 50 years and above as older adults in contrast to young adults aged 15–49 years who have been the focus of much of the HIV-related research (50). Therefore, to be eligible for this study, participants had to be at least 50 years old. Other inclusion criteria included to be residing in Chatsworth (an urban community) or Botha's Hill (a predominantly semi-rural community); being willing to participate; and ability to provide written informed consent. More crucially, only individuals who had participated in the phase one component were eligible for this qualitative component.

Some socio-demographic characteristics of individuals included in the qualitative interviews are displayed in Table 1. Mean age of study participants was 59.6 years (standard deviation 7.1). In total, 37 individuals (48.6% women) participated in the FGDs. Most study participants were black African (73.0%), currently married (67.6%), had a primary (48.6%) or higher (40.5%) level of education, and were receiving a government grant (73.0%). Furthermore, 67.6% said they were sexually active, and an estimated 10.8% were HIV positive.

**Table 1:** Characteristics of participants in focus group discussions.

**Characteristic**	**Category**	***n* (%)**
Participants		37 (100)
Mean (sd) in years		59.6 (7.1)
Sex	Women	18 (48.6)
	Men	19 (51.4)
Population group	African	27 (73.0)
	Non-African	10 (27.0)
Marital status	Never married	5 (13.5)
	Currently married	25 (67.6)
	Separated, divorced, or widowed	7 (18.9)
Education	Never been to school	4 (10.8)
	Primary	18 (48.6)
	Secondary or higher	15 (40.5)
Income	None	9 (24.3)
	Government grant	27 (73.0)
	Other	1 (2.7)
Sexually active	No	12 (32.4)
	Yes	25 (67.6)
HIV status	Negative	33 (89.2)
	Positive	4 (10.8)

### Design

This analysis uses data from a cross-sectional qualitative study conducted in 2017/2018 in Durban, South Africa. This was a follow-on study from an initial quantitative study conducted on the same population in 2016. Details of the initial study have been published elsewhere (51, 52). From the 435 individuals who participated in the initial study, we used a computer system to randomly select 37 individuals to participate in four focus group discussions (FGDs) across two sites. FGDs were conducted using semi-structured interview guides that elicited responses and experiences related to the following five broad domains: (1) general health concerns among older people; (2) norms and attitudes regarding sex, sexuality, and HIV in old age; (3) experiences of HIV in older people; (4) perceived behavioral control regarding sexual behavior; and (5) facilitators and barriers to accessing sexual health and HIV services. Follow-ups and probing questions were used in response to answers provided in the discussions. This helped to elucidate the context and meanings of the responses provided and created more open and dynamic discussions.

### Data collection

Potential participants for this study were given an introductory briefing on the qualitative component including the expected procedures to be performed. Individuals who expressed interest to participate in this part of the study were invited to the research site where they were fully informed about the study and undertook the informed consent process. Participation in the study was voluntary, and participants were informed that they could withdraw from the study at any point. After completion of the informed consent, participants were invited and scheduled for FGDs. Separate FGDs were conducted for men and women, irrespective of HIV status. Research assistants conducting the discussions were blinded to participant's HIV status and were well trained to avoid involuntary disclosure. All involved in the focus group discussions were asked to respect each other's privacy and maintain utmost confidentiality of the information shared. We used experienced and well-trained research assistants of the same gender as participants to conduct the interviews. The research assistants also received study-specific training including role-plays prior to facilitating the interviews.

### Data analysis

The FGDs were conducted in a quiet room at the site. Each FGD lasted approximately 70 min and was tape recorded. The recorded FGD audio files were transcribed verbatim by research assistants (SS, CB) into the language of the interview, which was isiZulu for all FGDs, except the female FGD at Chatsworth as all participants were of Indian origin and comfortable in English. The transcribed FGD files were translated into English and back-translated to the native language by experienced native language speakers. The transcripts were then quality controlled by a third person to ensure accuracy in the translations and back translations.

Data were analyzed by MN and JH-H and then discussed as a team for a more nuanced understanding of the results. Thematic analysis was followed using steps suggested by Braun and Clarke (53) and as recently applied by Fauk et al. (54) in a study of HIV transmission among a highly vulnerable group. These steps included (i) familiarization with the data by the researchers reading each transcript several times to immerse themselves in the data; (ii) generation of an initial or draft codebook based on the initial themes emerging from the transcripts; (iii) finalization of the codebook by connecting and comparing the codes from the data extracts.; (iv) review and refining of the themes and sub-themes through several review and consensus-building sessions; (v) defining and naming the identified themes, categories, and sub-themes out of the data; and (vi) extracting meaning and essence of each theme. A combination of inductive and deductive reasoning was used to extract meaning from the themes and sub-themes on the experiences of older persons with HIV prevention and care services in the community. Direct quotations were used to explicate participants' lived experiences.

The robustness of the study with respect to credibility, transferability, dependability, and confirmability, as per the ethnographic or phenomenological naturalistic paradigm inquiry, was achieved using several strategies (55, 56). These strategies included conducting the interviews in the local language to ensure participants fully understood the questions, audio recording of discussions to facilitate verbatim transcription of the discussions enriched by participants' expressions, having multiple debriefing sessions, and adopting a reflective attitude to minimize the researchers' bias (57). Furthermore, rigor and trustworthiness were also achieved by a thorough data analysis process, which included consultative sessions with community advisory boards.

All analyses were conducted using Nvivo version 12 (58), a software program used for the analysis of qualitative data.

### Ethical considerations

Ethical approval for this study was obtained from the South African Medical Research Council Ethics committee (EC030-9/2015). Participation in the study was voluntary. Written informed consent was obtained from the individuals for the publication of any potentially identifiable images or data included in this article. Pseudonyms were used throughout the focus group discussions and for all quotations used in subsequent reports. All participants in this study received reimbursement of R150 per FGD for loss of income, transport costs, and time spent at the research facilities as per standard practice in such research projects (59).

## Results

### Description of major themes emerging

Table 2 presents the summary of the major themes, categories, and subthemes emerging from this analysis. Experiences of neglect and abuse of older persons are presented in two phases. First, we describe the experiences of older persons not living with HIV in relation to accessing HIV prevention programs and services. Thereafter, we describe the experiences of older people living with HIV in accessing HIV care and treatment services.

**Table 2:** Themes, categories and subthemes of abuse and neglect of older persons.

**Theme**	**Category**	**Subthemes**
• Factors impacting HIV prevention and transmission in older people	• Sexual health and HIV information in the community	• Older people not well-informed about HIV
		• Challenges with accessing sexual health and HIV education in the community
		• Challenges with health worker involvement in community delivery of HIV education
• Individual and community responses to HIV and the abuse in older people	• Reactions to HIV in older people	• Fear and shame of HIV in old age
		• Difficulties of disclosure and discussing HIV in the community
	• Disrespect and shaming of OPLHIV	• Gossip and slander of OPLHIV
		• Loneliness and abandonment of OPLHIV
• Structural drivers of abuse in OPLHIV	• Challenges of staff attitudes and practices	• Inappropriate or disrespectful HIV services
		• Fraudulent and ageist attitudes
	• Challenges and consequences of disintegrated services	• Abuse of OPLHIV through involuntary disclosure
		• Resource and financial strain in accessing HIV care services

From the data, three major themes emerged as follows:

- Factors impacting HIV prevention and transmission in older people not living with HIV
- Community responses to HIV in older people contributing to abuse in older people
- Structural drivers of abuse in older people living with HIV (OPLHIV)

Details about these themes, categories, and subthemes are presented in the following section with appropriate quotations from the raw data to substantiate each theme.

## Experiences of neglect and abuse in older persons not living with HIV

### Factors impacting access to HIV prevention programs and services of older persons

#### Lack of information on HIV among older people

##### Not well informed about HIV

The results of this analysis showed that older persons were not well informed about HIV prevention measures. Older people reported not knowing how to live with HIV and the benefits of adhering to treatment should they become HIV infected. Participants highlighted a lack of health and HIV prevention messages targeted at older persons. This was described as a problem that worried and troubled older persons who desired to receive sexual health information and services:

“*You also must know that sometimes we feel like the older people are not very informed about a lot of health issues. They are always questioning, you know medical issues especially, they are not well informed*.” (Woman, 58, HIV-)

“*We are not well informed…what troubles or worry us about HIV/AIDS, …. we are not well educated about prevention methods from the virus.”* (Man 64, HIV-)

“*We must receive sex education because some of the old women are still dating, so it is important to get information.”* (Woman, 65, HIV-)

Older persons expressed worry and concern about HIV and wanted to be better informed about the risks of transmission and acquisition. A 52-year-old woman shared her fear of HIV acquisition in old age and associated it with shock and horror, saying HIV acquisition is “*a frightening experience, especially at our age*.”

“*For a whole woman, for a 50 -year-old women to have HIV, what must I say? I would say it's a story to the world…. “This lady is too old but look at her”*!”. (Woman 59, HIV-)

That is, participants considered HIV infection above the age of 50 years as a shocking experience. Participants explained that in the view of the community, one would be considered too old for HIV acquisition over 50 years. Therefore, they believed that being diagnosed with HIV infection in old age would certainly elicit negative community responses.

##### Challenges with accessing sexual health and HIV education in the community

During the group discussions, participants were probed about any places in the community where they could get information on sexual reproductive health (SRH) and HIV prevention services. These were services offered in the community outside a health facility. Many participants reported that there was no such place in their community. Some identified that there were SRH services in other areas, but not in their specific community.

“*There is no other place [to access to SRH and HIV information]*.” (Woman 51, HIV-)

“*No they [SRH information] are not available in this community, not at all, but I know of those in town. For instance, at Waterfall, you can consult with a nurse, talk about everything troubling you until you are satisfied*.” (Man 66, HIV-)

The need for sexual health and HIV prevention services for older people at community-based centers was clearly identified by participants not living with HIV in this study. Participants expressed a need for sexual health and HIV services to be delivered in their community. They specifically identified the need to cater to men and wellness.

“*The government should render these [SRH] services for the community, by building facilities, provide health care workers, have things such as Men's clinic, Wellness… Health care*.” (Man, 64, HIV-)

##### Challenges with health worker involvement in community delivery of HIV education

Another sub-theme regarding accessing HIV prevention services for older people not living with HIV reported by participants in this study related to the healthcare staff attitude, which was reported to be a major factor in determining whether HIV prevention services were provided to older persons in the community as well as the quality of those services. While participants living with HIV reported receiving information on “how to live with HIV,” participants not living with HIV revealed that HIV prevention information was only provided *ad hoc* depending on the healthcare workers' “mood.”

“*Yes, you are right that's how I know it, I got awareness and counseling on how to live with HIV but where I am no one talks about it, you don't get an awareness in your community, but you find it somewhere*.” (Man, 71, HIV+)

“*At the clinic, we also receive information [HIV prevention], when they are in a good mood they explain things in detail, especially in the morning. They remind us about the importance of using condoms.” (Woman 53, HIV-)*

Participants did not just want a place in the community where they could get information about sexual health and HIV, they also desired greater involvement of healthcare workers including doctors in community health outreach and HIV prevention programs. Other participants though reported that these community outreach health services are also an opportunity for other community leaders such as civic councilors to get involved in the delivery of HIV prevention services.

“*Doctors here in …. clinic they must involve themselves with the community in our halls and explain this thing [HIV] but it does not happen.”* (Man, 71, HIV+)

“*I was going to say that too, the councilors in the wards they are the ones who should counsel us about HIV, they must come to the community to do HIV counseling.”* (Man, 70, HIV-)

In this analysis, a factor that was a major contributor to perceptions of neglect and abuse among older people wanting HIV prevention services to be delivered in the community was poor staff attitudes. Participants reported that healthcare workers who came to their community on a weekly basis displayed a poor attitude toward community outreach services. Healthcare workers' attitudes were perceived to be worse at community health outreach programs than at the health facility. Participants reported that healthcare workers conducting community outreach services were arrogant and treated older people with disrespect. This made older people feel devalued and undermined.

“*You know on Tuesdays the staff who works at … clinic goes to the community, but when they arrive, they do not want to work as they do when they are at the clinic…. but now the way they treat us, we do not treat each other with respect. …they become arrogant, they undermine us*” (Man, 71, HIV +)

## Experiences of neglect and abuse in older persons living with HIV

### Community responses to HIV and abuse in older persons

#### Reactions to HIV acquisition by older people

##### Fear and shame of acquiring HIV in old age

Participants reported on experiences of living with HIV. Our analysis suggests older people living with HIV (OPLHIV) were better informed about HIV. OPLHIV reported that they even passed on their knowledge of HIV prevention to their children.

“*Yes, HIV I live with it, as I live with it what troubles me mostly is that… if I don't drink my tablets, I will get sick as I was counseled. I remind my children to use condoms always when they have sex and encourage my children to go for circumcision*.” (Man 71, HIV+).

##### Difficulties of disclosure and discussing HIV in the community

However, participants reported that HIV stigma and discrimination were common in the communities of the study population. Participants explained that community stigma and discrimination associated with HIV hampered disclosure in older persons, who would not talk about HIV, hide their HIV status, and ascribed HIV-related health issues to other diseases like diabetes and tuberculosis (TB).

“*Anything that I'm aware of where I live, they* [older people] *got HIV, but they don't speak about it (inaudible). The only thing they talk about is TB, but we know they got HIV, but they don't speak it out.”* (Woman 71, HIV-)

#### Disrespect and shaming of older people living with HIV

##### Gossip and slander of OPLHIV

Participants explained that OPLHIV face abuse in the form of gossip and slander. Participants reported that community members would discuss their status and how they may have acquired HIV behind their backs. Sometimes, this would include laughing about and ridiculing the OPLHIV.

“*People often talk behind your back, wondering how you got HIV as an old person*.” (Man 69, HIV-)

“*Like I said before, people talk behind your back, look at you, but it doesn't concern them, they should be helping instead of laughing at them. They discuss how the person got the virus, wonder who infected them, who brought the virus home between the couple.”* (Woman 65, HIV-)

A 71-year-old man described the discriminating practices in more detail. He reported that older people living with HIV are de-personated, isolated, judged, and discriminated against for living with HIV. He explained that based on these discriminating practices, he keeps his HIV status a secret and does not disclose it for fear of becoming the “laughingstock” of his community.

“*Can I talk, ehh in our community a person who is HIV*+*, they don't look at him as a person who think for himself, they judge and discriminate you if you have HIV. They see you as nobody, that's why I don't disclose that I take ARVs, and I hide it. I hide that so that nobody knows or find out. I make it my secret, because if somebody sees it the secret will be out and everybody in my community will know about it, we are taken like nobody, it's better to hide it when using the ARVs because you will be a laughingstock in your community, can I stop there for now.”* (Man, 71, HIV+)

Another participant concurred that people living with HIV in his community do not disclose their status. OPLHIV on antiretroviral treatment (ARVs) did not also want to disclose their usage of such medicines for fear of discrimination and isolation:

“…*in my community they do not come out those who are taking ARVs, it's not easy to know them, if it happens that we know the person who is taking ARVs, you are discriminated and isolated by people*.” (Man 70, HIV-)

Participants also revealed that persons living with HIV are called names such as *ibaqoqe* (loosely translated as those sick of HIV/AIDS) or *umqhakazo* (those who collect or are on treatment), which are considered derogative words in their community. As such, OPLHIV tend to isolate and associate among themselves.

“*Actually, people with HIV they talk about it with each other, it's not easy to know if they are available or who live like them.”* (Man 71, HIV-)

“*They hide those who have it [HIV]. If you are HIV positive, people always know that you are infected, they call it ‘*baqoqe' *and they will say you have “ibaqoqe”.”* (Man 71, HIV+)

HIV was also sometimes associated with supernatural influences. One participant explained that older people living with HIV were in some instances believed to be under the power or spell of the *sangomas* or ancestors.

“*A person lose weight, people say she has “amagobongo”* [meaning under the power of *sangomas* or possessed by the ancestors] *whereas they have this thing [HIV] but we heard that people who have this thing [HIV] are no longer dying. When a person has this thing [HIV], they say they have “amagobongo”. They use different reasons to cover up that you have it [HIV], they would say you have “amagobongo”, you have been bewitched or you ate something (idliso).”* (Woman 51, HIV+)

Participants revealed several concerns about potential stigma and discrimination. However, a 70-year-old man reflected that although he was aware of stigma and discrimination for people living with HIV in general, he did not know of anyone among his peers who has been discriminated against.

“*Maybe they are 3 or 4 people in my community that I know who are in the same age as I, but I haven't seen being disrespected or get discriminated.”* (Man 70, HIV-)

However, some participants urged older persons not to be ashamed of becoming HIV positive.

“*It's something that you shouldn't be ashamed of or not feel shame that it went to the extent that now we are positive with HIV*.” (Woman 52, HIV-)

##### Loneliness and abandonment of OPLHIV

Loneliness and abandonment of OPLHIV were other forms of abuse reported during the FGD. Participants experiencing loneliness reported their desire to share their life and experience with another person, in particular another person living with HIV. One lonely participant who had been abandoned by his wife expressed a desire to have somebody and be physically intimate with a fellow HIV-infected person.

“*I stay alone at home am not staying with any women since am diabetic and don't get erection. The women I had left me alone, she is now staying with another man. I wish to have someone who is also positive and on ART so that we can talk about our tablets. I want someone who we will remind each other on taking tablets when it's time. We can be happy by having sex and use condoms.”* (Man 71, HIV+)

## Structural drivers of abuse in OPLHIV

### Challenges of staff attitudes and practices

#### Inappropriate and disrespectful HIV prevention services

Participants described a lack of HIV prevention messaging in the health facilities that consider people of different age groups. The participants explained that their source of information on HIV prevention usually came from posters or flyers displayed around the clinics.

“*But when you go to the hospitals, clinics or something, you do see posters. So that is where we [get] information on this* [HIV prevention] *you know - use condoms and things like that, so that is how we educate ourselves by reading.”* (Female 58, HIV-)

Participants also reflected on the sexual health and HIV education offered in person at the health facilities. They reported that these sessions combined younger and older persons and sometimes the education sessions were delivered by younger persons, which older people experienced as inappropriate and disrespectful.

“*It happens that you find that awareness in the clinics if there is a person who is doing the awareness. They talk about it when we are mixed old and young people. As old people we want to be separated from youth, if you are there you will hear people talking about unprotected sex, or in the radio talking about how it's made.” (Man, 70, HIV-)*

“*I remember one day, I cursed a young girl who came to the clinic to teach people about female condoms, she had a rubber ring with her. She was demonstrating to people how the female condom is to be used. As she went on explaining and demonstrating about the female condom. I was not comfortable at all, it disturbed me a lot to see a young girl, who was educated but using her education to tell us something like that. It was going to be better if an old person was saying those things not a child. She was saying the right things, politely but we as old people perceived it as a disgrace or disrespect to talk like that.” (Man, 63*, HIV-)

“*I also remember when I was working at …. we had a 2-days course where we had to learn about the importance of condoms. A young girl was our instructor, we were very old, she could literally be my child. We had to sit and listen to her asking all sort of questions. She would ask us if we knew how to have sex? No, no, no...How does that sound, a child coming to teach old people? She could be a child to me, yet she was there to teach me. It means she was not raised well, but again she was sent by the government to come and teach us.” (Man 80, HIV-)*

Some participants extended this view around age to the research team and asked for the involvement of their peers in conducting the research.

“…*please use older people like myself when talking to older people…because you are young. You are not in a position to address old people. I am old I have knowledge that you do not have, …just because you went to school you think you can speak to different people, young and old but it is important to know that I am old and you are young*.” (Man, 63, HIV-)

Lack of age-appropriate HIV prevention information was described as causing worry in older people. Older people also described the emotional turmoil they experience in the form of feeling disgraced or disrespected when sexual health and HIV prevention services are delivered to them by young people or in the presence of younger people.

#### Fraudulent and ageist attitudes

Participants also reported on staff ageist attitudes that they faced when accessing HIV services at health facilities. For instance, a 70-year-old man reported that healthcare staff questioned why he wanted an HIV test when he was an old person. He explained that such ageist attitudes would discourage older persons to utilize HIV testing facilities.

*I once went for HIV testing and I was asked why the old person come for an HIV testing, so that will make us lazy to go and do the testing, the counselors must know that even old people they want to know about their health.”* (Man, 70, HIV-)

They believed that the “special treatment” of persons living with HIV could be due to bribery or enable health workers to pilfer medicines from the patients' packages.

“*I don't know, maybe there's something they give to nurses. I don't know maybe they are paying, yes those are packages for those who have a disease. We know it also happens to us.”* (Woman 53, HIV-)

“*They could take things for themselves from these packages.” (*Woman 65, HIV-)

“*The package has various medication; they take some and give the patient some.”* (Woman 53, HIV-)

### Challenges and consequences of disintegrated services

#### Abuse of OPLHIV through involuntary disclosure

Participants described the disintegration of health services at the healthcare facilities. Older people were critical of persons needing HIV services having their own queue or a separate building to access care. Where services were integrated, they reported that nurses used color-coded or different types of files for individuals in the queue for HIV services.

“*Yes, the only difference is HIV patients have got their own place, they've got their own clinic, my kind of thing now it's just general.”* (Woman, 60, HIV-)

“Special treatment” of people living with HIV was a contentious issue. Some older people believed their peers living with HIV should be given preferential treatment.

“*The HIV people are special, you know what I mean, we have to be kind to them, and you know we have to treat them well. To an HIV person you cannot pull our hand like, we need to pay attention to them and talk and love them and be polite to them*.” (Woman, 58, HIV-)

“Special treatment” also seemed to differ in different facilities or situations. For instance, one participant living with HIV said in his clinic there was no difference in how HIV and non-HIV patients were treated.

“*I don't get like personal treatment for that, I get treated just like every other patient, you know there is no special clinic, the best clinic is..., there is no more special unit for us, for people like who has all those (in-audible) so we get treated like a general patient.”* (Woman 56, HIV+)

Some older people were suspicious of the “designated areas” that were allocated to patients living with HIV. A 65-year-old man described how such structural factors were drivers of the enacted stigma, discrimination, and abuse experienced by OPLHIV.

“*There was a confidential thing that was shared here, we know that other people are not secretive at all if they see you in the container because they know that that area is designated for people who come to collect ARV's. You would soon hear people around the area, talking about you that you are on ARV's. You understand what I mean. This is so painful to hear rumors about you everywhere. HIV is seen as a bad thing... When a person sees me in those containers, they would perceive me in another way, maybe they would talk about me in the taxi.” (Man 65, HIV-)*

People not living with HIV complained that they were neglected compared to those “that have the disease (meaning HIV).”

“*In this clinic, they are very disrespectful, they don't even care what happens to old people, preferential treatment is given to people who come here to collect packages. I have had it with this clinic. We as old people need to come here and quickly go back home because we need time to rest. A young person needs to understand this, but they don't. Those who collect packages have that disease [referring to HIV].”* (Woman 57, HIV-)

The ability to provide feedback on health facility services seemed to be limited. Participants reported that they are not given an opportunity to raise issues at the health facility. Instead, they would be told to report to a well-known firebrand politician “Malema” to air their grievances, which they considered as rudeness or abuse toward them.

“*When people are angry about the system, they are told to call Malema, isn't that being rude to [older] people?”* (Woman 53, HIV-)

#### Resource and financial strain in accessing HIV care services

The separation of HIV services from other services at health facilities contributed to the abuse of older persons through involuntary HIV disclosure. Such experiences caused some older adults to seek treatment at facilities outside their communities, even at a great personal cost in time and money to themselves.

“*I get my treatment and go with it, I don't have a problem, don't want to collect it from the clinic* [near his home] *because they will talk, I always catch a taxi to here* [far from his home]*, they ask me always where do I have money to come here, I tell them that I don't have a problem, because am avoiding people talk that they had seen that am here to collect treatment.” (Man, 71, HIV*+*)*

Similarly, participants reported that the disintegrated services such as having separate rooms or files for collecting HIV medicine discourage some people to seek healthcare and resort to asking other people to collect medicines on their behalf or requesting to “borrow” medicines from other OPLHIV.

*We are all fetching our treatment, but some people think they are clever than others because they don't fetch their treatment, they send others to fetch it on their behalf. They are not shy to ask you to share with them our treatment. I normally ask them why they don't come and sit with the crowd to collect their medication? Why should I be a fool who gets shouted by the nurses at the clinic working for people? They give you their tickets* [clinic cards]. *Some people carry other people's tickets. A person is aware that they are sick, yet they still send people to fetch medication on their behalf. You see, I am comfortable, and I have accepted my situation. I take my treatment with no trouble at all. If I am coming here to fetch my medication, I openly tell them. You can be free to say anything you want, but in the end, you could also get it (HIV). They would say to me “please take my container to bring me my medication”. I just respond by saying, “why should I carry your medication when you are just sitting here?” (laughing). They would tell you to lend them your medication and they would pay you* [give back] *when they have collected it. I just tell them I can't because my medication would run out before time, and I wouldn't know how to answer nurses when asking me what happened to my medication. You remember that it was said that young boys are using this, they would assume that I am selling my ARV's*. (*Man 69, HIV*+*)*

Participants also reflected on some new initiatives that might overcome some of the negative experiences at healthcare facilities. This included medication delivery to their homes.

“*Now they are trying a new process of bringing us medication at home this would help us; we support this process it is the right thing. There won't be any reason for us to come and wait in queues until you are seen by people even your enemies whereas maybe he came to fetch tuberculosis medication and you are there to fetch HIV medication you become embarrassed.” (Man 65, HIV-)*

In summary, OPLHIV experienced certain practices at health facilities as stigmatizing, discriminatory and abusive when accessing care and treatment services. This included practices such as having a separate queue or separate building for HIV treatment, or identification of HIV status through color-coded or type of files. Participants who reported integration of HIV services with other health services reported less abusive instances.

## Discussion

In this qualitative study, we purposed to explore older people's experiences of accessing HIV prevention programs and services for those living without HIV and in accessing HIV care and treatment services for those living with HIV. We explored these experiences within a framework of neglect and abuse of older persons. Our study found that on the one hand, older persons living without HIV had limited knowledge of the HIV disease and ways in which to protect themselves, consistent with earlier reports on the impact of HIV on older persons (60). Thus, older persons without HIV were concerned and worried about HIV acquisition. They described it as a frightening experience and feared being judged and discriminated against for getting HIV in old age. This is in line with a similar study by Hlongwane et al. who explored how older adults navigated being diagnosed with HIV in communities in South Africa, which showed that older persons reacted with shock to an HIV diagnosis at their age (61). On the other hand, older persons living with HIV (OPLHIV) experienced a lot of community and health facility stigma and discrimination, similar to the negative social impact of HIV on other vulnerable groups such as women living with HIV in low- and middle-Income countries (62). The stigma associated with living with HIV may add to the mental distress among older people (31). Another finding of this study was that health facilities that separate HIV services from other health services were structural contributors to OPLHIV experiencing discrimination and the perpetuation of elder abuse. As recent evidence from Indonesia shows, persons living with HIV experience stigma and discrimination at multiple levels from family and community to healthcare settings (63).

This study found that part of the reason older persons without HIV may face HIV-related neglect and abuse is that they are not well informed about HIV due to the lack of older adults' targeted HIV prevention and care messages. Such information is usually available at health facilities, but older persons may have mobility challenges; hence, they are less likely to access these services. They may also not identify with the provided messages that often picture and target young people. That is why older persons without HIV in our study desired sexual health and HIV prevention services to be delivered in the community near to where they live by healthcare workers or even civic leaders. Similarly, Kelly et al. (64) who explored access to primary healthcare experiences of older people in Cape Town recommended community support services to enhance older people's access to healthcare (64). However, in the presented study, healthcare workers doing community support services were described as having a poor attitude in that they were arrogant and rude to older persons. The perception of participants in this study was that poor healthcare providers' attitudes were worse in the community than at the health facility level. Such treatment is a form of elder abuse that needs to be addressed through sensitization of healthcare workers and resource allocations to support older persons.

OPLHIV did experience psychological abuse in the form of loneliness and abandonment. Previous studies have found a close relationship between HIV-related stigma and loneliness (65). Data from a cohort of HIV-positive older persons from rural Uganda suggest some HIV-infected older persons may socially isolate themselves as a coping mechanism against stigma (29). Participants shared that stigma, discrimination, and disrespect toward HIV-infected older people were rife in their community. It was reported that older people who become infected with HIV are judged, discriminated against, and faced a lot of abuse in the community and at the health facilities. OPLHIV were considered less human, subjected to shame, and described using impolite words or names. As has been documented elsewhere, people living with HIV tend to experience discrimination from healthcare providers in the form of being criticized, being shouted at, having records thrown at their faces, being neglected, or being left without care (36, 66, 67). HIV-positive older people may also be accused of witchcraft and being possessed by evil spirits (68). This is supported by findings from an earlier study conducted in eight countries by the World Health Organization (69), as well as findings from other settings (36, 63, 66, 67).

This study found that there were some structural drivers of abuse, especially among OPLHIV accessing care services at health facilities. Participants shared experiences of accessing care in facilities with disintegrated services where those seeking HIV care and treatment have their own queues, separate building, or different types of files or color-coded files. Older people described such facilities as contributing to involuntary disclosure of HIV status in that those seen to be in those queues or buildings would instantly be known to be living with HIV and would be subjected to all the negative community responses to HIV in older persons highlighted earlier. The disintegrated services coupled with the anticipated stigma and discrimination from the community forced some participants not to seek HIV care and treatment services at health facilities within their local community, opting instead to travel to facilities outside their communities. As has been shown in other settings, many older persons face financial, transport, and long-distance challenges to access health services (61). Approximately 3.7 million people aged 60 years and older in South Africa receive a non-contributory old-age pension (70). This merger pension is in most cases used to support the entire household including adult children (71). Thus, older persons resorting to traveling long distances to escape stigma places a huge financial strain on them and their households. A recent South African policy encourages the integration of SRHR services (72). This is a step in the right direction in addressing the concerns of separate services and buildings. The practice of color-coded files needs some rethinking. On the one hand, color coding assist healthcare staff to triage patients and provide efficient care. On the other hand, color-coding practices can contribute to stigma. Patient folders should all look similar and instead small discrete codes that are only readable by healthcare staff should be used to support the triaging efforts for non-emergencies such as HIV.

Participants further shared experiences of poor healthcare worker attitudes and practices. Participants considered some HIV prevention programs at health facilities as inappropriate and disrespectful of older people in that older and younger persons would be mixed in the same session. Older persons in addition felt embarrassed and disrespected when the sexual health and HIV prevention sessions were delivered by younger persons. Participants described healthcare workers as rude and arrogant and felt disrespected for being made to wait a long time at the health facility. Healthcare workers were reported not to want to entertain older people's complaints about the health delivery systems. Some healthcare workers were also accused of fraudulent practices such as receiving bribes to fast-track some patients or pilfering some medicines from older people. There were also some ageist attitudes in that older people would be questioned for wanting an HIV test at their age—implying people of their age should not be having HIV. Such ageist stereotypes may contribute to delayed HIV diagnosis that has been reported for persons aged ≥50 (60). As shared by participants in this study, questioning older people wanting an HIV test may make them “lazy,” that is, make them reluctant to seek the service in future. This together with the already known low HIV risk self-perception among older persons (34, 61) is likely to make the uptake of HIV testing services by older persons extremely low. It is, therefore, imperative to address poor staff attitudes as well as their lack of awareness of the increasing risk of HIV in older persons aged ≥50 years (73). Attitudes and awareness can be addressed through training. For instance, the department of health could include an SRHR module focused on older people in their set of online SRHR modules (74). According to the department of health, the training modules targeting health providers are designed to achieve health for all but glaring leave out older people. There is a need for the development of older person-focused sexual health and HIV prevention messaging and programs, as suggested by Ellman et al. (60).

The poor healthcare worker attitudes and untoward practices toward older persons are tantamount to human rights violation and abuse. Action is, therefore, needed to ensure health workers deliver services as per their sworn oath to serve to their best “knowledge and ability for the safety and welfare of all persons” (75). Older persons in addition need to be empowered to claim their rights when they are denied by the poor attitude of health workers. As noted by the United Nations high commission for human rights and the joint program on HIV/AIDS, vulnerable populations including OPLHIV require support in accessing information on what rights they have and how to claim those rights (76).

It should be noted though that it was not only OPLHIV seeking HIV services who experienced abusive practices, even older people not living with HIV felt neglected and abused at health facilities. Participants expressed the view that much attention and special treatment is accorded to those living with HIV at the expense of those seeking non-HIV-related services. As evidence from the early years of the HIV pandemic shows (77), there was a reallocation of budgets toward HIV/AIDS expenditure from other services, which resulted in public health services being ill-equipped to offer long-term care to persons with other chronic conditions. The humongous resources invested in HIV care over the last two to three decades need to fertilize the care for these conditions now. This speaks again for integrated care that has already been initiated in South Africa with its integrated chronic disease management model (ISDM) (78). The ICDM model aims to leverage HIV programs and service delivery models to improve the quality of care for chronic diseases. The experiences of participants in this study suggest that integrated health services would also be experienced as less abusive by older people.

To address some of the challenges highlighted in this study with access to health services, there is a need to strengthen geriatric and nursing care training in South Africa. As reported by Frisoli (68) citing Priscilla Reddy in a report on the plight of South African older persons in relation to gerontological training (79), not a single institution in the country had a comprehensive course on gerontology. Elements of this specialty program were instead in various parts of training in medicine, sociology, and psychology. Nursing training is also in need of a holistic program in comprehensive care to older persons which incorporates older person's psychosocial needs to mitigate against elder abuse.

## Study limitations

This study used a qualitative design and is, therefore, limited with regard to its representativeness of the population of older persons in South Africa. The study also used research assistants who were significantly younger than the research participants. These assistants conducted the focus group discussions, which may have hindered complete and open discussion as alluded to by the participants who suggested that someone in their age group should conduct the focus group discussions. The authors also acknowledge the limitations of having combined persons living with HIV and those without in the same focus group. All participants were thoroughly informed that it would be a mixed group, and no one was obliged to disclose their HIV status. The mixed group might have encouraged the exchange of different ideas; however, it might also have restricted OPLHIV to share their own experience in the first-person account.

## Conclusion

We see from this study the indication of age stereotypes in the community and among older persons themselves in that there is a fear and stigma associated with acquiring HIV in older ages. Older persons appear to have internalized HIV discrimination and fear of getting HIV. This appears to be confirmed by the perceptions and actual experiences of older people living with HIV. Poor staff attitudes, color-coding files, and having separate facilities for HIV services are some of the main discriminating practices that participants identified. Although there were no reports of physical and sexual abuse of older persons in this study, there were clear cases of verbal, emotional, and even financial abuse among older people seeking HIV prevention and care services. This study shows that despite the many decades of HIV programming in South Africa, HIV-related stigma, discrimination, and disrespect of older persons remain pervasive in communities and at health facilities. Feasible interventions are thus needed to stymie experiences of disrespect and abuse of older people aging with and without HIV. The South African Department of Health's knowledge hub is one of the first opportunities to provide feasible training for healthcare workers across the country (80), but older people are not mentioned. As an increasing proportion of the population ages and lives longer with HIV, the neglect and outright abuse of older persons need urgent policy and program interventions in African settings.

## Data availability statement

The raw data supporting the conclusions of this article will be made available by the authors, without undue reservation.

## Ethics statement

Written informed consent was obtained from the participants for the publication of any potentially identifiable data included in this article.

## Author contributions

MN, SS, CB, and JH-H: study conceptualization, writing original draft, editing drafts, analysis, critical revision of manuscript, and approval of final article. MN, SS, and CB: data collection and curation. MN: resources. All authors contributed to the article and approved the submitted version.
